# Biopsychosocial Determinants, Diet Quality, Gastrointestinal Health, and Disease Activity in Adults With Rheumatoid Arthritis: Cross-Sectional Descriptive Study

**DOI:** 10.2196/79889

**Published:** 2026-01-08

**Authors:** Maureen McGarrity-Yoder, Emily K Cope, Heidi A Wayment, Alicia Rodriguez-Pla, Tracy E Crane

**Affiliations:** 1 College of Nursing Northern Arizona University Flagstaff United States; 2 Department of Biological Sciences Northern Arizona University Flagstaff, AZ United States; 3 Department of Psychological Sciences Northern Arizona University Flagstaff, AZ United States; 4 Sierra Pacific Arthritis and Rheumatology Centers Fresno, CA United States; 5 Department of Medical Oncology, Miller School of Medicine University of Miami Miami, FL United States

**Keywords:** diet quality, gastrointestinal health, gut microbiome, inflammation, rheumatoid arthritis

## Abstract

**Background:**

Rheumatoid arthritis (RA) causes pain, fatigue, joint deformity, disability, and an increased risk for serious sequelae, often despite treatment, in 1.3 million Americans. RA is affected by numerous biopsychosocial determinants, which greatly complicate treatment, including altered efficacy.

**Objective:**

The purpose of this study is to examine associations between individual biopsychosocial determinants, diet quality, gastrointestinal (GI) health, and disease activity in adults with RA.

**Methods:**

This cross-sectional, descriptive study has been approved by the Northern Arizona University Internal Review Board (# 2111208-12). We will include 96 adults with RA recruited from across Arizona using social media and community events (through the Arthritis Foundation) and various primary care and rheumatology practices in Flagstaff and the greater Phoenix metro area. Individual biopsychosocial factors will be measured with a demographic survey and direct measures. The Arizona Food Frequency Questionnaire will measure dietary intake for the past 6 months, and Healthy Eating Index-2020 scores will be calculated from these data. The Automated Self-Administered 24-hour diet recall will measure recent dietary intake. Fecal analyses for gut microbiome diversity and composition and fecal calprotectin will measure current GI health. Disease activity will be measured by the Health Assessment Questionnaire–Disability Index and pain scale, Disease Activity Score of 28 Joints, and hematology results (C-reactive protein and erythrocyte sedimentation rate). In addition to descriptive statistics, hierarchical linear regression will examine hypothesized associations between diet quality, GI health, and disease activity. We hypothesize that individual biopsychosocial determinants will be associated with diet quality, which will be indirectly associated with disease activity through gut microbiome diversity and level of GI inflammation in adults with RA.

**Results:**

This study was funded in February 2024. As of December 19, 2025, a total of 80 individuals have been recruited. Data analysis has not yet commenced at the time of manuscript submission. Study results are expected to be published in fall 2026.

**Conclusions:**

RA is a complicated disease that impacts millions. Few individuals reach sustained remission, even while following provider recommendations. A better understanding of the various factors that impact this complicated disease has the potential to support changes in research and care that will improve the lives of people with RA. The knowledge gained in this study will provide a foundation to inform future interventional research targeting diet quality to support GI health and decrease RA disease activity. Further, the details of this research plan provide methodological resources for other RA researchers, and research results have the potential to improve communication between rheumatology providers and patients.

**International Registered Report Identifier (IRRID):**

PRR1-10.2196/79889

## Introduction

### Background

Rheumatoid arthritis (RA) is a debilitating autoimmune disease that affects approximately 1.3 million Americans [1]. RA is characterized by intermittent, chronic inflammation that causes decreased physical function [2]. Treatment typically focuses on pharmacologic management to reach the goal of remission, but, unfortunately, many patients do not reach despite following provider recommendations [3]. Increased RA disease activity can result in decreased quality of life due to chronic pain, fatigue, and disability [4], impacting family, social, and work life [5]. Prolonged inflammation often results in bone and joint damage, deformity, altered psychological and social function, and an increased risk of serious sequalae such as hypertension, depression, and cardiovascular disease, again, often despite treatment [1,4,6,7]. RA also causes a high financial burden due to excessive medical costs, disability, and a decreased ability to work [4].

The exact etiology of RA remains uncertain, but it is understood that an underlying genetic risk is activated in the presence of an environmental stimulus [8]. The activation and progression of RA, characterized by intermittent local and systemic inflammation, ultimately results in joint deformity and bone breakdown if the disease is not well managed [8]. Localized RA inflammation includes redness, warmth, decreased function, pain, and edema of affected joints [9]. Systemic inflammation, measured with hematologic proinflammatory biomarkers [9], is often associated with neurological manifestations through the bidirectional neuroimmune loop. These manifestations are categorized as “sickness behavior”: fatigue, malaise, nausea, and withdrawn behavior [7,10,11]. Systemic inflammation in RA is also associated with poor sleep quality [12], mood changes and depression [13], and an increased risk of several comorbidities including mental health disorders (eg, anxiety and depression), interstitial lung disease, hypertension, and cardiovascular disease [7,8]. The objective of this funded project is to examine associations between individual biopsychosocial determinants, diet quality, gastrointestinal (GI) health, and disease activity in adults with RA. The long-term goal of this research is to improve rates of sustained remission to allow better health outcomes and quality of life for all diagnosed with RA.

### The Biopsychosocial Model of Disease Activity in RA

**Figure 1:**
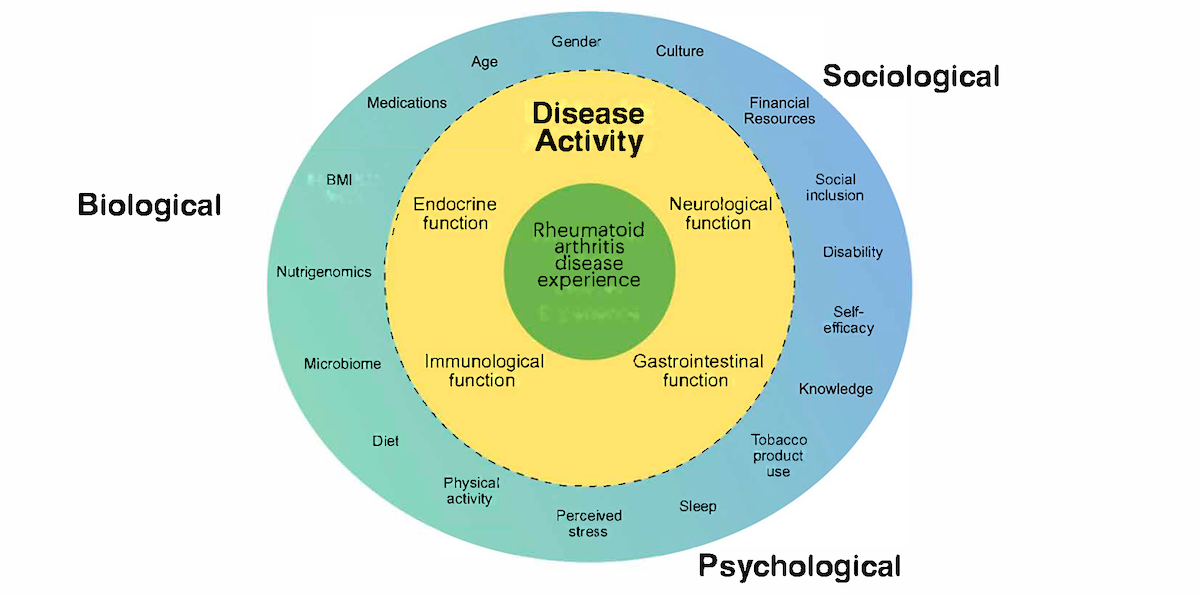
Biopsychosocial Model of Disease Experience in Rheumatoid Arthritis (BDRA) [1].

RA inflammation is impacted by various biological, psychological, and sociological factors. The evidence-based Biopsychosocial Model of Disease Experience in Rheumatoid Arthritis (BDRA) (Figure 1) considers these determinants of disease activity in RA. The BDRA, developed by the primary author [14], combines concepts from Engel’s Biopsychosocial Model of Health [15] and the Revised Symptoms Management Conceptual Model [16] to demonstrate these influential relationships in RA to better inform research and patient care. This model serves as the theoretical underpinnings of the proposed project and considers determinants individual impact; their effects on one another; and their impacts on neurological, immunological, endocrine, and GI function; and, finally, disease activity and experience in RA.

### Diet, Gut Microbiome and Diversity, GI Inflammation, and RA Disease Activity

There is significant patient interest in how diet may be related to disease activity in RA [17]. Although a few studies have reported associations between dietary intake and generalized inflammation, these associations are unclear, possibly due to the unreliable nature of self-reported dietary intake [18,19]. The ambiguity in the literature may also contribute to inadequate communication about diet recommendations between rheumatology providers and patients [20]. To date, researchers have yet to consider the effect of biological data to explain associations between dietary intake and RA disease activity. GI health (gut microbiome composition and diversity and GI inflammation) may be mechanistic underpinnings to explain these potentially important associations. Diet is known to impact the composition of the gut microbiome, and individuals with RA are more likely to exhibit gut dysbiosis, which is an imbalance of gut microbiota [21-23]. Gut dysbiosis has also been associated with RA development [24]. Fecal calprotectin, a protein released by neutrophils in the presence of inflammation, has been associated with various autoimmune diseases, including spondyloarthritis [25]. Further, increased serum calprotectin levels are associated with increased disease activity in RA [26].

### Biopsychosocial Factors

#### Overview

Background biopsychosocial factors known to be associated with RA disease activity include gender, age, socioeconomic status (education and household income), ethnicity, perceived stress, BMI, smoking, and medication use. Women are 3 times more likely to develop RA and have higher disability rates [27,28]. Functional disability and poor outcomes increase with age [28-30]. Worsened health outcomes in RA have been associated with poverty, decreased education, and a lack of basic health care services [31]. RA rates are significantly higher in minority populations [32]. Perceived stress has significant biological effects on disease activity through psychoneuroimmunological mechanisms [7,33]. Increased BMI and smoking are associated with higher rates, worse disease activity, and poor outcomes in RA [34,35]. RA medications disrupt various inflammatory pathways of the immune system [36].

#### Socioeconomic Status

Lower socioeconomic status, underserved communities, racial and ethnic minority groups, and sexual and gender minority groups are at risk for less health care access according to the National Institutes of Health (NIH) [37]. As with other disease processes, these individuals are at risk for poor outcomes in RA. Worsened RA outcomes [31] and decreased rates of remaining under rheumatology care have been associated with poverty [38,39]. Individuals with RA are at risk for social exclusion, which is often worsened with low socioeconomic status [31,40]. Increased disease activity in RA can further increase financial burden due to high medical costs and a decreased ability to work [4,31].

#### Gender

Women are 3 times more likely than men to be diagnosed with RA [27] and report worse pain and fatigue and exhibit greater evidence of disease activity [41]. Women are more likely to experience decreased productivity and missed work [42] and have higher rates of long-term functional disability [28].

#### Age

Functional disability [28] and rates of serious sequelae increase with age [30]. Further, increased rates of RA diagnosis occur in older adults [30].

#### Ethnicity

Individuals of color are noted to have higher rates of RA diagnosis [32]. Additionally, individuals of color also experience higher rates of poor outcomes [32,43].

#### BMI

Obesity is significantly associated with increased rates of RA [34]. Obesity also results in inflammation, worse disease activity, and poorer clinical outcomes [44].

#### Smoking

Cigarette smoking increases the risk for RA [45] and is associated with worsened disease severity, increased joint damage, and risk for sequalae (eg, malignancies, atherosclerosis, and vasculitis in RA) [35].

#### Medications

Medications used to treat RA include nonsteroidal anti-inflammatories, corticosteroids, and biologics (conventional, biologics, and Janus Kinase-inhibitors). They decrease inflammation by disrupting various inflammatory pathways associated with RA [36].

### Potential Biopsychosocial Factors: Diet and GI Health

GI health may be the bridge needed to clarify potential associations. Diet is known to impact the composition of the gut microbiome, and individuals with RA are more likely to exhibit gut dysbiosis [21-23]. Further, gut dysbiosis has been associated with RA development [24]. Fecal calprotectin, an indicator of GI inflammation, has been associated with disease activity in various autoimmune diseases, including spondyloarthritis [25], and increased serum calprotectin levels were associated with increased disease activity in RA [26].

#### Diet

Dietary metabolites can directly affect cellular production of proinflammatory and anti-inflammatory cytokines through signaling pathways [46]. Proinflammatory signaling pathways are directly stimulated by metabolites of refined grains and omega-6 polyunsaturated fatty acids and directly inhibited by metabolites of antioxidants and omega-3 polyunsaturated fatty acids [46]. Cancer, hypertension, heart disease, and type 2 diabetes are associated with a typical Western diet, characterized by high sugar, fat, protein, and salt content [8]. Cyclic citrullinated peptide antibody, a key biological mediator in RA, has been found to react with dietary triggers (eg, food and microbial proteins) and induce RA symptoms [47].

#### The GI Tract and Immune Function

The GI tract plays a significant role in immune function. Approximately 70% of lymphocytes in the human body are located within the GI tract [9]. Symbiotic and pathogenic organisms, located throughout the GI tract cause an almost constant potential for disruption of gut homeostasis [48]. The heavy presence of immune cells assists in protection against toxins and pathogens [48]. The GI tract further influences immune function through the brain–gut–microbiome axis, impacting the inflammatory response [48].

#### The Gut Microbiome

The gut microbiome is a key component of health and disease and significantly impacts immune function [49]. Patients with RA were found to have a significantly different composition and associated dysbiosis of gut microbiota compared with healthy controls [50]. This included an increased quantity of *Prevotella*, which is generally associated with GI mucosal inflammation and an increased production of proinflammatory cytokines [49]. Individuals in preclinical stages of RA were found to have an abundance of *Prevotella* spp, supporting the involvement of intestinal dysbiosis in the development of RA [51]. Patients with RA were found to have increased *Alloprevotella*, *Parabacteroides*, and *Prevotella-2*, which were previously associated with increased systemic inflammation in other diagnoses [24]. Interestingly, disease-modifying antirheumatic medications partially restore eubiosis, or a normal balance of gut microbiome composition, in patients with RA [52]. Lastly, gut microbiome dysbiosis and associated GI tract damage may result in passage of inappropriate molecules and microorganisms, referred to as a “leaky gut” [53]. This may be a mechanism through which dietary intake alters disease activity of various diseases, including RA. Interestingly, DNA from gut bacteria has been found in synovial fluid of patients with RA and healthy control patients, although in differing amounts based on RA diagnosis [54]. This offers preliminary evidence that the gut microbiome may impact the synovial microbiome and disease activity in RA.

#### GI Tract

GI inflammation is closely associated with gut microbiome balance. Calprotectin is a damage-associated molecular pattern protein released by neutrophils in the presence of inflammation. It is measurable in both serum and fecal samples. Fecal calprotectin indicates acute inflammation of the intestines and is significantly associated with other measures of inflammation in inflammatory bowel disease and spondyloarthritis [25]. Although fecal calprotectin has not been compared to disease activity in RA, serum calprotectin levels have been associated with increased disease activity in RA [26].

**Figure 2:**
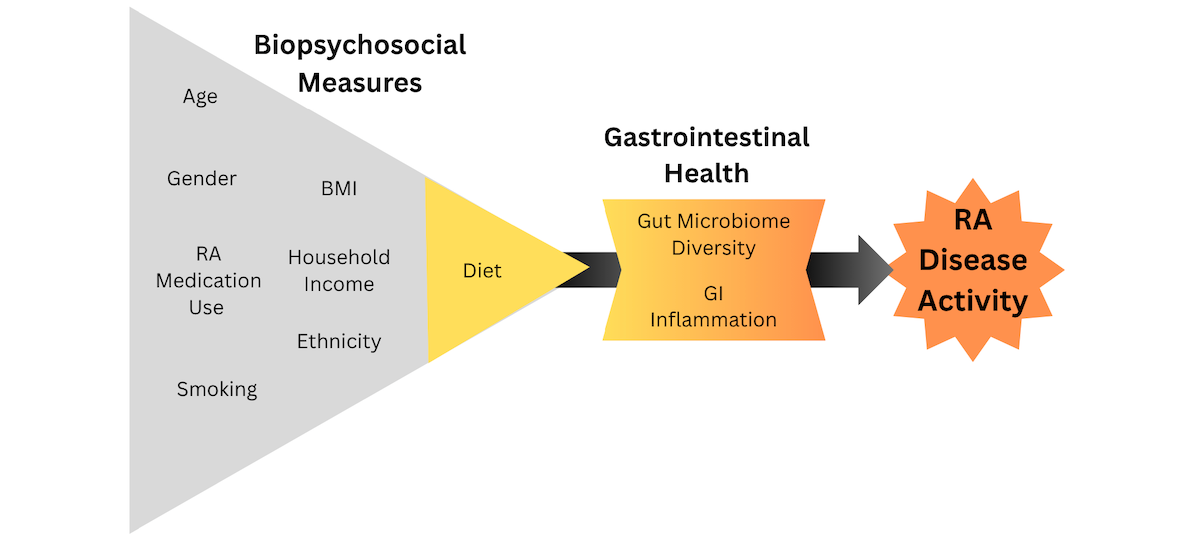
Study model. GI: gastrointestinal; RA: rheumatoid arthritis.

The proposed study fills an important gap in the literature by examining how background factors and biological measures of GI health (gut microbiome diversity and GI inflammation) may improve our understanding of the hypothesized relationship between dietary intake and RA disease activity (Figure 2).

### This Study

In the southwest United States, approximately 28,000 Arizonans are diagnosed with RA, including many Black, Hispanic, and Native American individuals. The rate of RA diagnosis is higher in women, and this rate increases for women of color. In Hispanic populations, for example, this rate is doubled compared with White populations [32]. Additionally, the prevalence of RA has been found to be significantly higher for Native Americans [43]. Further, poverty rates are high for many Native Americans. The Navajo Nation, for example, has a reported poverty rate of 38% [55], which is significantly higher than the Arizona average of 16.4% [56]. Unfortunately, RA studies continue to focus on individuals of European ancestry. This is a serious concern for patient outcomes as well as for the development and implementation of personalized medicine focused on genome-specific care [32]. The lack of diversity in RA research leads to a significant breach in patient care.

There are two aims for this study:

- Aim 1: Examine associations between diet quality, other important individual biopsychosocial factors, and GI health in adults with RA. Hypothesis 1: We expect individual biopsychosocial factors will be associated with diet quality, and most participants will report dietary intake that is either poor or fair quality per preliminary Healthy Eating Index (HEI)–2020 scores. We also expect that individuals who report lower diet quality will have associated decreased GI health.
- Aim 2: Examine predicted associations between diet quality, gut microbiome diversity and composition, and GI inflammation on RA disease activity. Hypothesis 2: We expect that diet quality will not be directly associated with RA disease activity; instead, we will find a significant indirect relationship between diet quality and RA disease activity through relationships between diet quality and biological measures of GI inflammation and gut microbiome diversity, and between gut health measures and RA disease activity. We will test our hypotheses with 4 indicators of RA disease activity. Past research has shown that the Disease Activity Score using 28 joint counts (DAS-28), HAQ-DI: Health Assessment Questionnaire–Disability Index (HAQ-DI) and Pain Scale, Erythrocyte Sedimentation Rate (ESR), and high-sensitivity C-reactive Protein (hsCRP) are moderately correlated. Our hypotheses regarding the role of gut health will be reinforced if either hypothesis is supported.

## Methods

### Research Design

This study uses a cross-sectional design to describe associations between background factors and diet quality and to test 2 a priori hypotheses regarding the relationship between diet quality, gut microbiome diversity, and GI inflammation on disease activity in RA. The inclusion of GI measures addresses an important gap in the literature and inform future research.

### Sample and Recruitment

**Figure 3:**
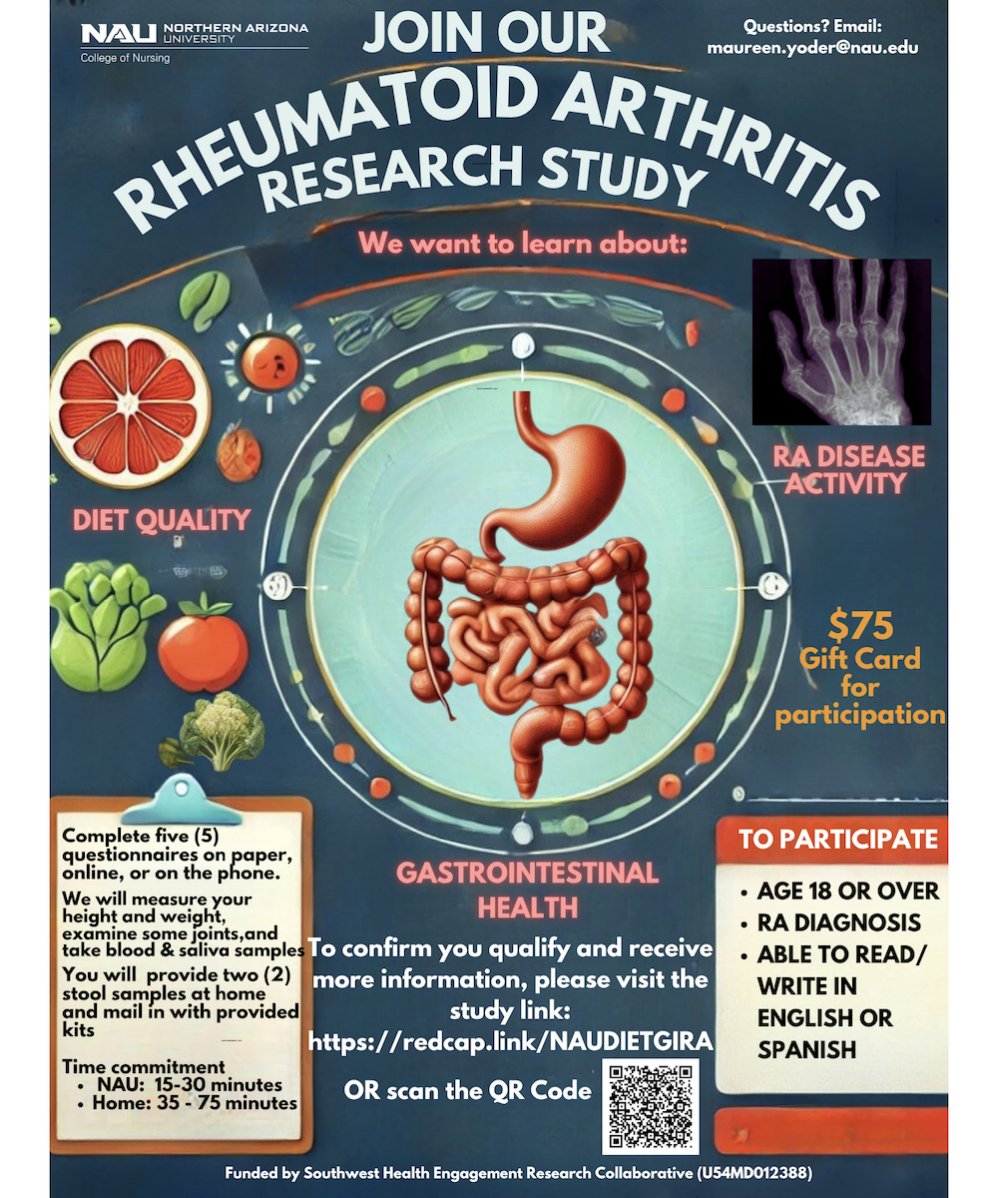
Recruitment poster. NAU: Northern Arizona University.

**Figure 4:**
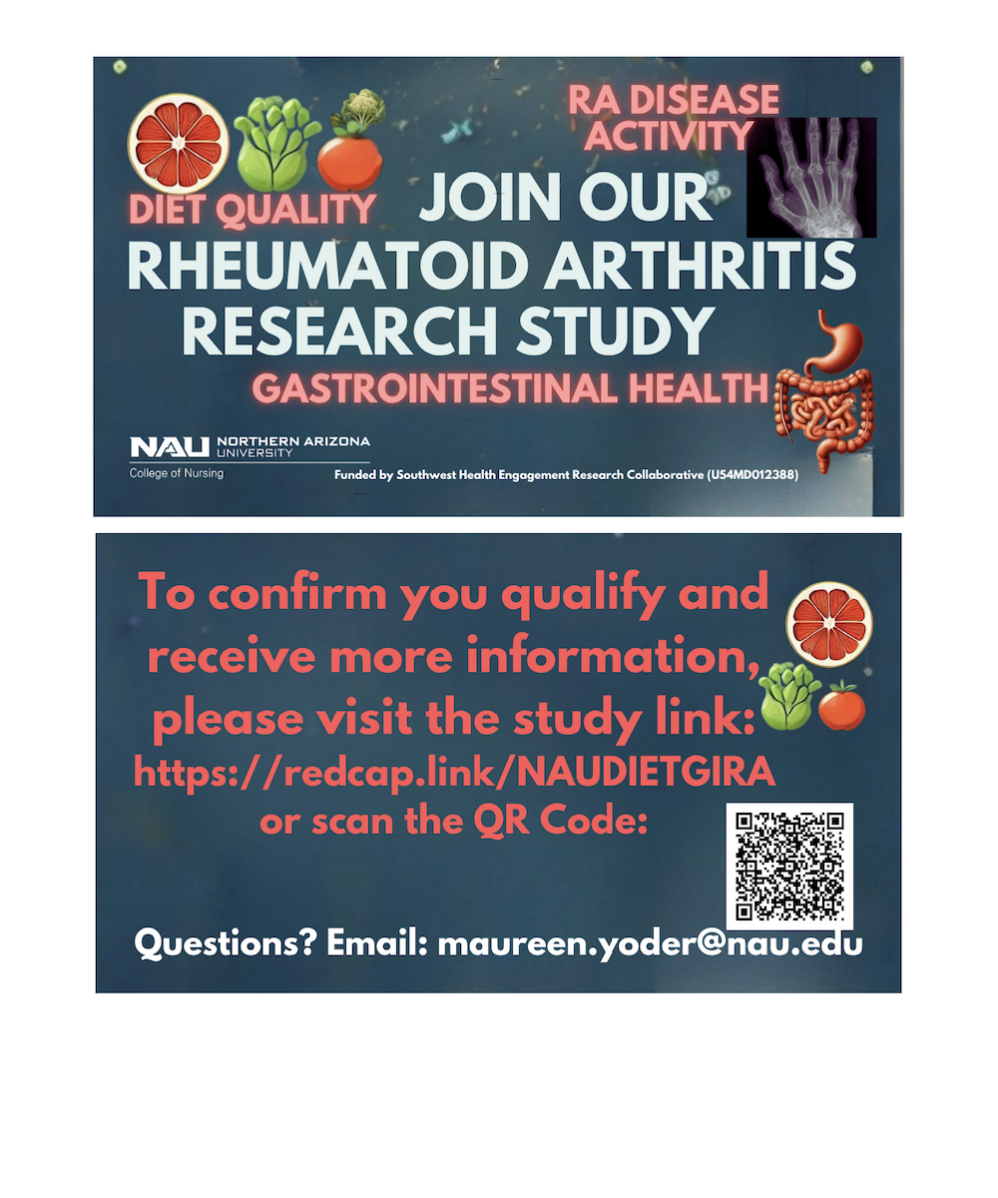
Recruitment business card.

**Figure 5:**
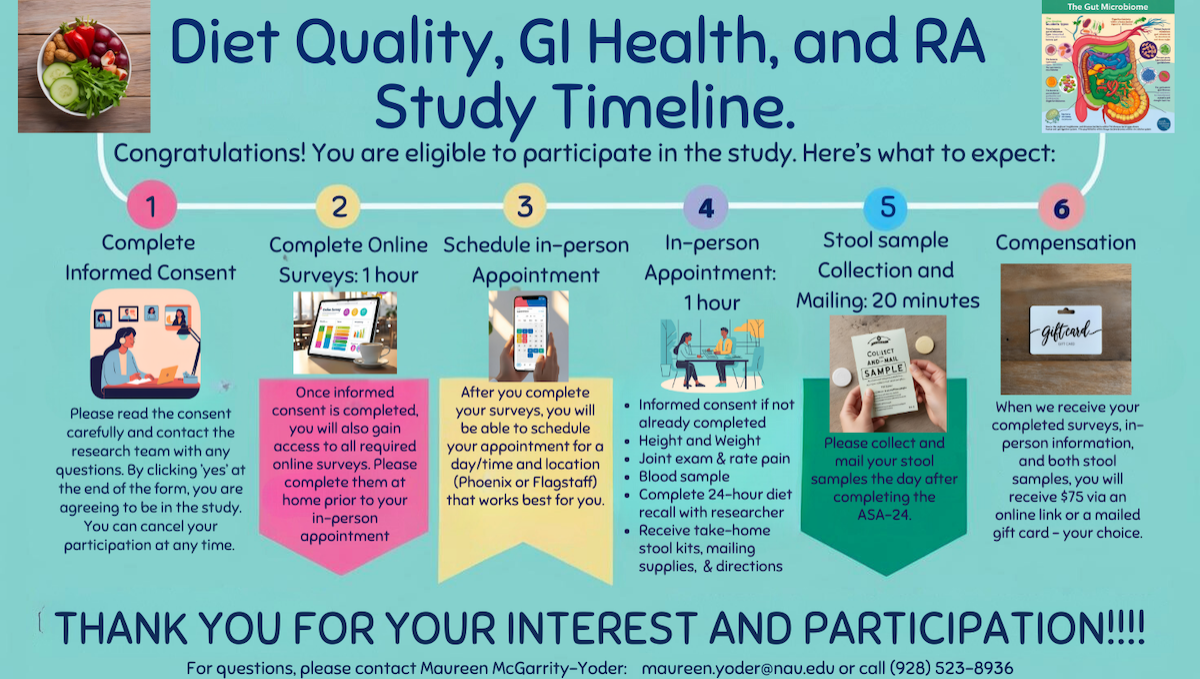
Study timeline for participants. RA: rheumatoid arthritis.

The target population for this proposed study are adults aged ≥18 years diagnosed with RA. We will use posters and business cards (Figures 3 and 4) to recruit adults with RA from a variety of locations including the Arizona Arthritis and Rheumatology Associates offices in Arizona (Flagstaff, Prescott, Avondale, Sun City, Glendale, Phoenix, Mesa, Gilbert, San Tan Valley), the Mayo Clinic Collaborative Research in Rheumatology (Scottsdale), Northern Arizona University, North Country Healthcare with locations in northern Arizona (Flagstaff, Bullhead City, Grand Canyon, Kingman, Show Low, Payson, Holbrook, Williams, Kingman, Round Valley, Winslow, Lake Havasu City), and the Arizona office of the Arthritis Foundation (including social media posts on Instagram and Facebook and at the Arthritis Run). The posters and business cards include a link to a website and a QR code that directs interested individuals to a secure eligibility survey through REDCap (Research Electronic Data Capture; Vanderbilt University). Eligible individuals will receive access to an online informed consent form. They will be directed to carefully read the informed consent form and will have the option to click “yes” to agree to participate in the study or “no” to decline. If they choose “no,” they will receive a message thanking them for their time and consideration, as well as information directing any questions or concerns to the principal investigator. Consented participants will receive access to all online surveys and timeline (Figure 5). Upon completion of all online questionnaires, participants will receive a link for the final survey to sign up for an in-person appointment on a convenient date, time, and location (Northern Arizona University Flagstaff or Phoenix North Valley Campuses). We will also offer specific days focusing on Spanish-speaking individuals. Inclusion criteria are that participants must be aged ≥18 years, able to read and write in English or Spanish, and have a diagnosis of RA that is managed by a rheumatology health care provider. Exclusion criteria for this study include age <18 years, pregnancy, inability to read or write in English or Spanish, or a diagnosis of any other autoimmune disease.

**Figure 6:**
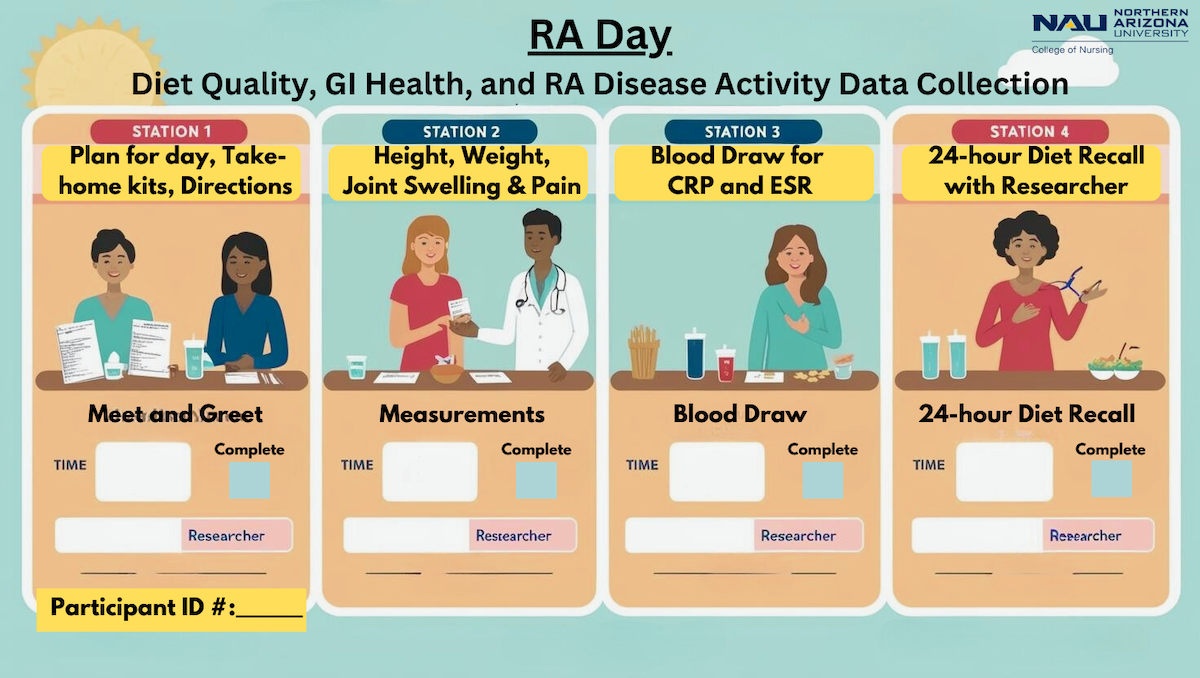
Checklist for data collection day. CRP: C-reactive protein; ESR: erythrocyte sedimentation rate; RA: rheumatoid arthritis.

Each data collection venue will be set up with 4 stations, beginning with a welcome table. On the appointment day, participants will first meet with the principal investigator or trained research assistant and receive a tote bag with instructions, stool collection kits, and an addressed, stamped shipping envelope for self-collection of stool samples. At this station, participants will also receive all directions to complete the study at home. Next, a researcher will work with the participant to measure height and weight, followed by the DAS-28, a physical exam to determine the number of swollen and tender joints, and pain level. Next, a blood sample will be drawn to measure ESR and C-reactive protein (CRP) levels, followed by meeting with a research assistant to complete the Automated Self-Administered 24-Hour Dietary Assessment Tool (ASA24), a dietary assessment. Figure 6 shows the informational checklist to be provided to participants.

### Data Management

Quantitative data will be examined for completeness as they are received. All data will then be cleaned and checked for errors before data analysis. Quantitative data will be analyzed descriptively using frequencies and proportions for the total sample. For nonnormal distributions of continuous data, medians and IQRs will be used. Categorical data will be presented using proportions and frequencies.

### Data Analysis Plan

Data from the above-described measures will be combined into a single dataset with any identifying information removed before analysis. All data analyses (and visualizations) will be conducted with SPSS (version 29; IBM Corp) and R (version 4.2.3; R Foundation for Statistical Computing). Before analyses data will be cleaned and examined for outliers, and on the basis of distributions, appropriate data transformations will be computed to meet assumptions for parametric statistical tests. If not, nonparametric tests will be performed where needed. For Aim 1, we will conduct appropriate parametric or nonparametric statistical tests with self-report measures to examine how our primary model variables are differentially associated with background factors. Bonferroni-adjusted *P* values will be used to control for Type I errors. In our analyses, we will use the HEI-2020, HAQ-DI/Pain Scale, and DAS-28 scales as continuous measures. For ease of comparison with existing literature, we will recode these measures into the published categorical designations to describe our sample. In addition to the examination of *P* values, CIs will also be reported. This study will use a hierarchical linear regression to test 2 separate hypotheses that diet quality will be indirectly related to RA disease activity through its expected relationships with gut diversity and gut inflammation. Each model will regress one of the RA disease activity variables onto the following measures: step 1, previously identified significant demographic characteristics; step 2, continuous measure of diet quality; and step 3, two measures of GI health.

### Power

To determine sample size, several multiple regression power analyses (fixed model, *R*^2^ deviation from zero), were completed using G*Power software (version 3.1.9.6) [56]. The analyses were calculated using power specified at 0.80 across 3 different effect sizes (f^2^) and 3 different α levels (0.05, and 2 types of Bonferroni-adjusted values) [57]. Two effect size approximations were based on published correlations on measures similar to those proposed in this study (diet quality, GI inflammation, gut diversity) with DAS-28 (f^2^=0.120) and HAQ-DI/pain (f^2^=0.083) measures, and one effect size (0.15) was a general recommendation for a medium effect [58]. We determined that a sample size of 96 would be a reasonable sample size to test our two study hypotheses (f^2^=0.12; power=0.80; α=0.05) and slightly higher than the sample size of 91 determined using a conservative α with Cohen medium effect size (f^2^=0.15; power=0.80; α=0.025). A post hoc power analysis (fixed model, *R*^2^ increase) also indicated that a sample size of 96 is adequate to insure at least 80% power to detect the ability of our two primary predictor variables (GI inflammation and gut diversity) to contribute at least 8% additional variance in our outcome measures (RA disease activity) above and beyond the variance (20%) expected to be predicted by our 6 background measures.

### Measures

#### Background Measures

Background measures will be assessed with a demographic questionnaire that includes questions regarding yearly household income (less than US $25,000, US $25,000-US $49,999, US $50,000-US $74,999, US $75,000-US $99,999, US $100,000-US $124,999, more than US $125,000, do not wish to answer, or do not know for certain), sex (male or female), age, zip code years since diagnosis of RA, preferred language for communication, highest educational level (elementary, high school or General Educational Development, vocational training, some college or associate’s degree, bachelor’s degree, master’s degree, doctoral degree, or do not wish to answer), marital status (single, married, separated, divorced, widowed, other, or do not wish to answer), race (American Indian or Alaska Native, Asian, Black or African American, Native Hawaiian or other Pacific Islander, White, more than one race, other, or do not wish to answer), current tobacco use, current cannabis use, 2 questions regarding food security, and current medication use (disease-modifying antirheumatic agents, corticosteroids in the last month, nonsteroidal anti-inflammatories in the last week). The demographic questionnaire also includes questions regarding dietary beliefs associated with RA symptoms.

#### Diet Quality

The Arizona Food Frequency Questionnaire (AFFQ) will be used to measure dietary intake. The 175 item self-reported AFFQ is a modified version of the National Cancer Institute Health Habits and History Questionnaire and will be used to determine self-reported intake (foods, portion sizes, beverages, and vitamins and minerals) for a 4-week period [57]. Reliability of the AFFQ demonstrated a correlation of 0.63, and test-restest reliability correlation of 0.48 (adjusted for energy-related nutrients) and 0.54 (unadjusted nutrients) [57]. The AFFQ will be used to determine participant intake (foods, beverages, and portion sizes) to estimate intake for a total of 34 variables [57]. The AFFQ is available in English and Spanish. Diet quality will be scored with the HEI-2020, which is the most recent update of the HEI based on the comparison of dietary intake to the 2015-2020 Dietary Guidelines for Americans [58,59]. The HEI-2020 was developed in a collaborative effort of the National Cancer Institute and the US Department of Agriculture to score diet quality based on comparison of dietary intake to the 2015-2020 Dietary Guidelines for Americans [58,59]. The HEI-2020 is scored out of 100 points: less than 51 is poor, 51-80 is fair, and above 80 is good diet quality [9]. Americans have an average score of 59 out of 100 [60]. AFFQ analysis and HEI-2020 score calculations will be completed at the Behavioral Measurement Intervention Shared Resources at the University of Arizona.

The ASA24 will be used to measure dietary intake for the previous 24-hours. The self-reported ASA24 was initially based on the previously validated Automated Multiple-Pass Method [61], and this validated, reliable tool has been used to complete over 1 million 24-hour diet recalls, with collected data used in over 1000 peer-reviewed publications [62]. Various usability and cognitive tests have been completed on sequential versions of the ASA24, and indicate similar results to interview-administered, standardized 24-hour diet recalls [63]. Research has indicated that approximately 80% of consumed items are reported with the ASA24 [64]. The ASA24 is available in English and Spanish.

#### Gut Microbiome

Fecal microbiome composition and diversity will be assessed using 16S rRNA gene sequencing. Sequencing will be performed by the Translational Genomics Research Institute North Pathogen and Microbiome Division in Flagstaff, Arizona. 16S rRNA gene sequencing is one of the most widely used applications to examine bacterial microbiota [65] and is commonly used in RA research [66-72]. Stool samples will be self-collected at home by participants, who will receive a toilet accessory, disposable gloves, a sterile specimen cup, an OMNIgene GUT DNA RNA Fecal Collection Tube, 2 biohazard bags, 1 shipping box, and 1 plastic FedEx Clinical Pak. Fecal samples will be collected in OMNIgene GUT Fecal collection kits, which stabilize DNA and RNA for transport at room temperature for several days. Participants will place the samples in the appropriate shipment box, enclose each with the plastic FedEx Clinical Pak, and attach the appropriate shipping label. The samples must be shipped via FedEx Monday through Wednesday. Samples will be stored at the Pathogen and Microbiome Institute at Northern Arizona University in Co-I Cope’s lab at −80°C until transported to and analyzed at the Translational Genomics Research Institute in Flagstaff. Samples will be extracted using a Kingfisher Flex system that extracts 96 samples in a single batch and sequenced on the same Illumina MiSeq run. If interim analyses are necessary, we will include 5 replicate samples on each run and mock communities to assess batch effects.

#### Microbiome Bioinformatics

Microbiome bioinformatics will be performed with QIIME 2 (Quantitative Insights into Microbial Ecology version 2) [52] using Denoising Algorithm Directed by Amplicon Variants for sequence quality control and definition of amplicon sequence variants to provide the highest possible taxonomic specificity. Alpha diversity (community richness) metrics will be computed with QIIME 2, including Faith’s Phylogenetic Diversity, observed operational taxonomic unit, and Shannon diversity. Beta diversity (community dissimilarity) metrics will also be computed with QIIME 2, including Bray-Curtis dissimilarity, Jaccard, weighted UniFrac, and unweighted UniFrac distances. These diversity metrics, along with taxonomic profiles of samples, will be used to compare microbiome compositions associated with categorical variables. Group comparisons of alpha diversity will be performed with nonparametric Kruskal-Wallis tests, and group comparisons of beta diversity will be performed with nonparametric PERMANOVA. Since identifying amplicon sequence variants and taxa that are differentially abundant across disease groups is currently a very active area of research, we will assess available methods at the time of analysis for performing this step but expect to work with multiple methods, including established methods such as analysis of compositions of microbiomes with bias correction. All *P* values will be corrected for multiple comparisons using the Benjamini-Hochberg false discovery rate correction.

#### GI Inflammation

GI inflammation will be determined with fecal calprotectin levels using enzyme-linked immunosorbent assay. Fecal calprotectin values in adults of less than 50 µg/g are normal, 50-100 µg/g indicate moderate inflammation, and greater than 150 µg/g indicate significant inflammation [73]. Samples will be collected and shipped as described for the gut microbiome analysis. Samples will be assessed using the Human IL-6 enzyme-linked immunosorbent assay kit by Sigma Aldrich (RAB0306) as per manufacturer’s instructions.

#### Health Assessment Questionnaire–Disability Index and Pain Scale

The HAQ-DI and Pain Scale will be used as a self-reported measure of RA disease activity, as it is frequently used in research and clinical practice [74]. The HAQ-DI and Pain Scale is recommended by the American College of Rheumatology [74]. It was chosen due to participant experience, low burden, and ease of scoring [75]. The HAQ-DI and Pain Scale is a 9-item self-report (8 items associated with HAQ-DI and 1 item for pain) that determines the overall health or disease activity of patients with RA [74,76]. Test-retest correlation has been reported as 0.87-0.99, and HAQ-DI score had a correlation of 0.71-0.95 with task performance [75]. Validity of the HAQ-DI has been found “in literally hundreds of studies” [75]. Total scores range from 0 to 3: where 0-1 indicates mild to moderate disability, 1-2 indicates moderate to severe disability, and 2-3 indicates extremely severe disability [74].

#### Disease Activity Score Using 28 Joint Counts

The DAS-28 is frequently used in practice and research, as it offers ease of scoring and low participant burden [77]. The DAS-28 is a valid, reliable tool that uses hematologic biomarkers, examination, and participant-reported information to calculate disease activity. DAS-28 CRP and DAS-28 ESR scores have been highly correlated (*r*=0.92; *P*<.001) [78], and this project will use the DAS-28 ESR, as was used in a recent study [79]. The DAS-28 has had high internal consistency reliability (Cronbach α=0.719) and is significantly correlated with disability as measured by a health assessment questionnaire (*P*<.001) in other work [77]. Preliminary work offered evidence of significant correlation between erythrocyte sedimentation levels, CRP levels, and DAS-28 scores; therefore, DAS-28 scores will be used as evidence of inflammation in the proposed project. Scores of <2.6 indicate remission, scores of 2.6-3.2 indicate low disease activity, and scores >3.2 indicate moderate to severe disease activity [80].

#### Hematologic Biomarkers

Hematologic proinflammatory biomarkers will be used as evidence of disease activity in this study, as they are a direct measure of inflammation. These biomarkers included the ESR and hsCRP analyses. The ESR indicates generalized inflammation by the rate of erythrocyte sediment that accumulates in a test tube over 1 hour; the normal range for adults is 0-20 mm per hour (male) and 0-30 mm per hour (female) [81]. The hsCRP indicates generalized inflammation, as CRP is an acute-phase protein produced by the liver in response to increased levels of interleukin-6 in the blood; values of less than 1.0 are normal, 1.0-3.0 indicate moderate inflammation, and values greater than 3.0 indicate high levels of inflammation [7,81]. While the ESR and hsCRP indicate generalized inflammation in the body, not specific to RA, both are used to determine severity of disease activity in patients with RA [81].

### Ethical Considerations

This study has been approved by the Northern Arizona University Institutional Review Board (2111208-12). Eligible participants will receive online access to the informed consent form in REDCap in their choice of English or Spanish. They will be directed to carefully read the form and click “yes” to agree to participate in the study, or “no” to decline. If they choose to participate, they will receive access to all online surveys (their choice of English or Spanish); at the end, they will be directed to self-schedule an in-person appointment. Those who choose not to participate will receive a message of thanks for their time and consideration. Participants can withdraw from the study at any time. There are no risks associated with participant withdrawal. As this is a cross-sectional study, participants who choose to withdraw will not be asked to continue in any other activities related to this research study.

Researchers will use the secure REDCap, Qualtrics, and NIH platforms for participant questionnaires. Each participant will receive an online link or code to access online platforms. Questionnaires will be uploaded into REDCap, with the exception of the AFFQ, which is only available in Qualtrics, and the ASA-24, which is located on a secure NIH website. Questionnaires collected on paper will have the participant ID code placed in the top right corner of each document. All questionnaires, including the demographic questionnaire, will remain nonidentifiable. Stool and hematology samples will be labeled with participant ID number only. Nonidentifiable data will be securely stored on an encrypted, password-protected drive. The principal investigator will have access to the data. Non–personally identifiable data will be kept indefinitely for future analyses.

Once the completed surveys and both stool samples are received, participants will be sent a US $75 gift card (either an electronic or physical card, based on participant preference).

## Results

This study was funded in February 2024. As of December 19, 2025, a total of 80 individuals have been recruited. Data analysis has not yet commenced at the time of manuscript submission. Study results are expected to be published in fall 2026.

## Discussion

### Overview

Biopsychosocial factors such as socioeconomic status, age, ethnicity, gender, BMI, smoking, and RA medication use are known factors that can affect eating habits and therefore may influence the gut microbiome, but this research question has not yet been examined in patients with RA. Our exploratory analyses will examine the extent to which background variables are associated with dietary quality and biological markers of gut health, including GI inflammation and gut microbiome composition and diversity, known to be altered in individuals with RA [24]. We have reasonable expectations that low socioeconomic status will be associated with poor diet quality, and most participants will report dietary intake that is either poor or fair quality per preliminary HEI-2020 scores [17]. We also expect individuals reporting low diet quality will have associated gut microbiome dysbiosis and higher fecal calprotectin levels, indicating increased inflammation [21-23,26]. Significant results from analyses will be used in subsequent analyses to account for patterns related to diversity of experience and background in patients with RA. There is little evidence that self-reported diet quality, is a reliable predictor of RA disease activity. Preliminary work completed by the primary author examined associations between diet quality and disease activity in 50 adults with RA. Diet quality scores were lower in participants (average score 56) compared to the national average (average score 59) [79]. Women (*P*=.003) and those with increased age (*P*=.02) were more likely to report a higher diet quality [79]. A total of 44% of participants reported that they believe their diet affects RA disease activity, and these participants were significantly more likely to change their diet (*P*<.001). Higher education level (at least some college) was associated with this belief (β=–1.535; *P*=.02). Participants with lower diet quality had higher pain (β=–0.396; *P*=.02) and inflammation (*P*=.02) [79]. Significantly higher HAQ-DI scores, an indicator of disability, were noted in women (β=.570, *P*=.001) [79]. Although decreased levels of proinflammatory biomarkers were previously associated with a higher quality diet [80], no other associations were found between diet quality and overall disease activity [79]. This lack of evidence may be accounted for by the diet quality scores of participants in this preliminary work, as only 4% (n=2) individuals exhibited “good” diet quality [79]. Other considerations are that this was a relatively homogeneous (74% White) sample. Diet quality may directly impact functional ability (eg, shopping, preparation, and even eating food) in RA [9].

By including biological markers of gut health (presumed to be affected by diet quality), we will be able to test 2 hypotheses regarding how gut health may be a key link to understanding the relationship between diet quality and disease activity in patients with RA. We expect that diet quality will not be directly associated with RA disease activity; instead, we will find a significant indirect relationship between diet quality and RA disease activity through relationships between diet quality and biological measures of GI inflammation and gut microbiome diversity, and between GI health measures and RA disease activity. Our hypotheses regarding the role of gut health will be reinforced if either hypothesis is supported. To the best of our knowledge, this project is the first to consider associations between background biopsychosocial variables, diet quality, GI health, and disease activity in adults with RA. An innovative advantage specific to this study is the comparison of an existing, vetted self-reported dietary measure with measures of GI health.

### Possible Limitations and Alternate Solutions

Obstacles may occur with data collection. Participants may have difficulty completing paper or online questionnaires if they have significant pain or deformity of their wrists, hands, or fingers. To address these concerns, participants will have the option of a telephone interview for all surveys. A further obstacle may be participant recruitment, as a total of 96 participants are needed. A total of 55 participants were previously recruited at one rheumatology office in 50 days. The sample previously recruited was homogeneous, as 68% reported some form of higher education (at least some college), and 74% identified as White. To address this, we will recruit from across Arizona using social media through the Arthritis Foundation, as well as from rheumatology practices across the greater Phoenix metro area, which, according to the research manager, serves a diverse and underserved community.

### Conclusion

RA is a complicated disease that impacts millions [14]. Few reach sustained remission, even while following provider recommendations [3]. A better understanding of the various factors that impact this complicated disease has the potential to support changes in research and care that will improve the lives of people with RA. We believe biopsychosocial factors will be associated with self-reported diet quality, which will be indirectly associated with disease activity through gut microbiome diversity and level of GI inflammation in adults with RA. The knowledge gained in this study will be a foundation to inform future interventional research targeting diet quality to support GI health and decrease RA disease activity. Further, the details of this research plan provide methodological resources for other RA researchers.

## Appendix

Multimedia Appendix 1 Peer review report from the Pilot Project Program (PPP) Award Committee, Investigator Development Core, Southwest Health Engagement and Research Collaborative (SHERC) at Northern Arizona University.

## Abbreviations

AFFQ: Arizona Food Frequency Questionnaire
ASA-24: Automated Self-Administered 24-Hour Dietary Assessment Tool
BDRA: Biopsychosocial Model of Disease Experience in Rheumatoid Arthritis
CRP: C-reactive protein
DAS-28: Disease Activity Score using 28 joint counts
ESR: Erythrocyte Sedimentation Rate
GI: gastrointestinal
HAQ-DI: Health Assessment Questionnaire–Disability Index
HEI: Healthy Eating Index
hsCRP: high-sensitivity C-reactive Protein
NIH: National Institutes of Health
QIIME 2: Quantitative Insights into Microbial Ecology version 2
RA: rheumatoid arthritis
REDCap: Research Electronic Data Capture

## Data Availability

The datasets generated and analyzed during this study will be available in the OpenKnowledge@NAU repository at Northern Arizona University.
