# Supplementary material for: Biopsychosocial Determinants, Diet Quality, Gastrointestinal Health, and Disease Activity in Adults With Rheumatoid Arthritis: Cross-Sectional Descriptive Study
Source: JMIR Res Protoc. 2026 Jan 8;15:e79889. doi: 10.2196/79889 (PMC12828320; doi:10.2196/79889)
Supplement: Multimedia Appendix 1 [file resprot_v15i1e79889_app1.pdf]

SHERC Pilot Project Proposal Review

Application Title: Is it a gut feeling? The impact of socioeconomic status, diet quality, and gastrointestinal health on disease activity in rheumatoid arthritis

Principal Investigator(s): M. McGarrity-Yoder

Reviewer #: 1

OVERALL IMPACT

Please provide an **overall impact score** to reflect your assessment of the likelihood for the project to yield a sustained, meaningful contribution to the research field(s) involved. Prior to providing your Overall score, please provide a score and develop bulleted strengths and weaknesses statements for the five criteria: Significance, Investigators, Innovation, Approach and Environment. If you do not have comments for the strengths and weakness, stating *None noted* is acceptable.

Overall Impact *Write a paragraph summarizing your review. What is your overall impact score? 4. Between 1- 9 with 1 being the best score and 5 being average.*
This study proposes to examine the impact of biopsychosocial factors, specifically socioeconomic status (SES), diet quality, gut microbiome composition and gastrointestinal composition inflammation on disease activity in adults with RA. The proposal is grounded in the existing literature and proposes to build a foundation for understanding the role of multiple factors to turn, inform interventions or clinical trials that might assess the impact of alternative therapies for RA patients, beyond medication. The strengths of the proposal are the clear research design, the partnering health care facility to assure the projected sample size can be attained and PI's collaborators and mentors who bring needed experience in gut microbiome composition, diet-cancer associations, as well as the pathogenesis of complex rheumatic diseases. The primary weaknesses of the proposal are in sample recruitment and the somewhat junior status of her collaborators/mentors. With attention to health disparities, low income is the primary variable of diversity. The PI's previous work has been with predominantly Caucasian sample; the contribution to health disparities research is questioned.

n

Impact	Score	Descriptor	Additional Guidance on Strengths/Weaknesses
High	1	Exceptional	Exceptionally strong with essentially no weaknesses
	2	Outstanding	Extremely strong with negligible weaknesses
	3	Excellent	Very strong with only some minor weaknesses
Medium	4	Very Good	Strong but with numerous minor weaknesses
	5	Good	Strong but with at least one moderate weakness
	6	Satisfactory	Some strengths but also some moderate weaknesses
Low	7	Fair	Some strengths but with at least one major weakness
	8	Marginal	A few strengths and a few major weaknesses
	9	Poor	Very few strengths and numerous major weaknesses

SCORED REVIEW CRITERIA

Please consider each of the five review criteria below to determine merit. Please give a separate score for criteria.

1. **Significance** Should this research be done?

Does this project advance our knowledge and understanding of achieving health equity?

Y*our score?* ***4****. Between 1- 9 with 1 being the best score.*

Strengths

- The information gained in this proposal could significantly expand the understanding of factors that contribute to increased disease activity in adults with rheumatoid arthritis.
- The study design will lead to assessing the role of four predictors of disease activity (SES, diet quality, gut microbiome diversity and GI tract inflammation) and five confounding variables (gender age, body mass index, smoking status and use of RA medication). Identifying the drivers of RA disease activity with attention to the confounding variables could guide dietary interventions to treat RA.

Weaknesses

- A primary weakness of this proposal is the lack of attention to racial/ethnic diversity. In the background information the proposal does report higher RA prevalence in minority populations, yet this study does not propose to expand the scientific understanding of RA disease activity and health disparities related to race/ethnicity.
- The proposal does address the impact of low SES on diet quality, but the current design does not describe efforts to engage even an economically diverse population.

2. **Investigator(s).** Will it be done?

Is the team qualified to accomplish the tasks identified? Do they have relevant background and experience? Remember this funding is intended for early-stage investigators who may not have a long career record but should have expertise in the area.

Y*our score?* ***4.*** *Between 1- 9 with 1 being the best score.*

Strengths

- The PI has demonstrated expertise exploring alternative treatments, e.g., diet and gut microbiome health, in autoimmune disease, specifically rheumatoid arthritis (RA).
- In the past several years, she has provided national and international presentations on the biopsychosocial determinants of the disease experience in RA.
- The PI has assembled collaborators, Drs. Cope, Rodriguez-Pla and Trane, who bring needed expertise to this research, specifically microbiota composition or function influence on human disease, pathogenesis and epidemiology of complex rheumatic disease and cancer control and prevention through dietary and physical activity intervention.

Weaknesses

- The team member support on this proposal is minimal, only the PI and one graduate research assistant working 5 hours/week for 40 week are covered by the budget. The proposal identifies a number of tasks, including recruitment, participants training, data

collection and analysis and dissemination; all activities will be completed by the PI. The PI may have underestimated the time needed for these activities and the associated travel, as recruitment extends beyond Flagstaff. Insufficient personnel may negatively impact study progress.

- Consultants and mentors are not supported on the proposal. This restraint may limit the time Dr. McGarrity-Yoder's collaborators may have to work with her.

- Regular collaborator/mentor meetings are not described, so not clear how often and the content of the guidance received from her colleagues. The collaborators although extremely productive and accomplished are relatively junior; the challenges present by academic status, time and their own need for productivity, are not clear.

3. **Innovation.** Is the idea sufficiently novel, that it should be done?

Will the methods or outcomes provide new information?

Your score? 3. Between 1- 9 with 1 being the best score.

Strengths

- Previous RA research has not examined multiple biopsychosocial factors influencing the efficacy of RA treatment. The innovation of this research has the potential to build a strong foundation to support a clinical trial designed to assess the impact of alternative treatment therapies for RA patients.

Weaknesses

- The project would benefit from innovative recruitment strategies. Currently, recruitment will occur at one rheumatology office in Flagstaff, the University of Arizona Arthritis Center in Tucson, and Arizona Arthritis and Rheumatology Associates, who have offices in Flagstaff, Phoenix and Tucson. The PI acknowledges the previous recruitment with the Flagstaff office yielded a 74% Caucasian sample.

- Establishing partnerships with facilities that have a high minority patient population, would have supported the recruitment of an ethnically diverse sample.

4. **Approach**. Can it be done?

Are the steps the research team is proposing understandable? Do the steps seem to make sense to address the problem identified?

Your score? 4. Between 1- 9 with 1 being the best score.

Strengths

- The study is built on the existing literature and experience of the investigators and offers a clean design to examine biopsychosocial factors influencing RA disease activity.

Weaknesses

- The validation of the data collection instruments should be provided, specifically the AFFQ and Health Assessment Questionnaire Disability Index (HAQ-DI) and Pain Scale. The citations provide examples of instrument use in previous studies but does not speak to validation with heterogenous populations.

- The results of the study may be difficult to interpret; the sections addressing analysis could be expanded. Not sure if analysis will require a biostatistical consultant.
- Dissemination allowance was allocated to scientific dissemination, not community dissemination. Offering a presentation(s) for RA support groups could be one means to reach non-scientific audiences and not exclusively providers.
- The study proposes to examine the influence of income as related to diet quality, yet recruitment strategies do not ensure that low income and/or minority individuals will be included in the participant sample.

5. **Environment.** Will it be done, thinking about the time investigators are investing and equipment available to them.

Does the research team describe resources that will ensure they can accomplish the goals of the project?

*Your score? **3**. Between 1- 9 with 1 being the best score.*

Strengths

- The academic/research environments and health care partners are adequate to conduct this research.
- Dr. McGarrity-Yoder has received professional development support from her home department and provides a strong support letter from her Chair.

Weaknesses

- Expansion of health care facility partners would support this study providing insight to the health disparities of RA disease activity.

ADDITIONAL REVIEW CRITERIA

As applicable for the project proposed, reviewers will consider the following additional items in the determination of scientific and technical merit, but will not give separate scores for these items**.**

— Responses for Protections for Human Subjects, Vertebrate Animals, and Biohazards **are required from reviewers for all applications**.

— A response for Inclusion Plans is required from reviewers for applications proposing Human Subjects Research, except those designated Exemption 4.

Study Timeline (Specific to applications designated clinical trial on the electronic cover sheet)

Strengths

- Time allocated to individual activities is feasible.

Weaknesses

- Research group meetings are slated to occur throughout the two years of the project; a statement of the frequency of these meeting would have demonstrated consistent consulting and mentoring.

Protections for Human Subjects
Acceptable Risks and/or Adequate Protections<br>Comments (Required Unless Not Applicable):<br>•<br>Data and Safety Monitoring Plan (Applicable for Clinical Trials Only):<br>Acceptable<br>Comments (Required Unless Not Applicable):<br>○

Inclusion Plans **Applicable Only for Human Subjects research and not IRB Exemption #4.**
• Sex/Gender: Distribution justified scientifically<br>• Race/Ethnicity: Distribution not justified scientifically<br>• For NIH-Defined Phase III trials, Plans for valid design and analysis: Click Here to Select<br>• Inclusion/Exclusion Based on Age: Distribution justified scientifically<br>Comments (Required Unless Not Applicable):<br>•

Vertebrate Animals
Is the proposed research involving vertebrate animals scientifically appropriate, including the justifications for animal usage and protections for research animals described in the Vertebrate Animals section (and method of euthanasia described in the Cover Page Supplement or PHS Supplemental Form, if applicable)?<br>Not Applicable (No Vertebrate Animals)<br>Comments (Required Unless Not Applicable):<br>•

Biohazards
Acceptable<br>Comments (Required Unless Not Applicable):<br>•

Resubmission
Comments (if applicable):<br>•

Renewal

Comments (if applicable):
•

Revision
Comments (if applicable):<br>•

ADDITIONAL REVIEW CONSIDERATIONS

As applicable for the project proposed, reviewers will address each of the following items, but will not give scores for these items and should not consider them in providing an overall impact/priority score.

Applications from Foreign Organizations
Not Applicable (No Foreign Organizations)<br>Comments (Required Unless Not Applicable):<br>•

Select Agents
Not Applicable (No Select Agents)<br>Comments (Required if Unacceptable):<br>•

Resource Sharing Plans
Acceptable<br>Comments (Required if Unacceptable):<br>•

Authentication of Key Biological and/or Chemical Resources
Acceptable<br>Comments (Required if Unacceptable):<br>•

Budget and Period of Support
Budget Modifications Recommended (in amount/time)<br>Recommended budget modifications or possible overlap identified:<br>• To ensure the timely engagement of consultants/mentors and collaborators resources might be allocation to support these investigators.

SHERC Pilot Project Proposal Review

Application Title: *Is it a gut feeling? The impact of socioeconomic status, diet quality, and gastrointestinal health on disease activity in adults with rheumatoid arthritis.*

Principal Investigator(s): Maureen McGarrity-Yoder, PhD

Reviewer #: 2

OVERALL IMPACT

Please provide an **overall impact score** to reflect your assessment of the likelihood for the project to yield a sustained, meaningful contribution to the research field(s) involved. Prior to providing your Overall score, please provide a score and develop bulleted strengths and weaknesses statements for the five criteria: Significance, Investigators, Innovation, Approach and Environment. If you do not have comments for the strengths and weakness, stating *None noted* is acceptable.

Overall Impact *Write a paragraph summarizing your review. What is your overall impact score?*____5_______*. Between 1- 9 with 1 being the best score and 5 being average.*
This proposal aims to study the association between a range of factors (SES, gut microbiome, gastrointestinal inflammation and diet) and Rheumatoid Arthritis disease outcomes (Health Assessment Questionnaire-Disability Index/pain scale and the Disease Activity Score of 28 Joints). The project is moderately novel and uses validated outcomes, which are described in detail, demonstrating knowledge of prior research. The study will also focus on recruitment of women from a range of diverse backgrounds disproportionally affected by RA and relatively understudied. The project feasibility is further enhanced by the PI's experience with recruitment and engagement with clinical partners and given that the protocol only requires one measurement and approximately 80 participants. The investigative team is very strong, although only the PI is supported on the proposal, leaving questions about capacity for data analysis and microbiome assessment. A moderate limitation is related to the proposal emphasizing the complexity of the interrelated factors, but proposed analysis evaluate each factor separately (also, no conceptual or theoretical framework is mentioned).

Impact	Score	Descriptor	Additional Guidance on Strengths/Weaknesses
High	1	Exceptional	Exceptionally strong with essentially no weaknesses
	2	Outstanding	Extremely strong with negligible weaknesses
	3	Excellent	Very strong with only some minor weaknesses
Medium	4	Very Good	Strong but with numerous minor weaknesses
	5	Good	Strong but with at least one moderate weakness
	6	Satisfactory	Some strengths but also some moderate weaknesses
Low	7	Fair	Some strengths but with at least one major weakness
	8	Marginal	A few strengths and a few major weaknesses
	9	Poor	Very few strengths and numerous major weaknesses

SCORED REVIEW CRITERIA

Please consider each of the five review criteria below to determine merit. Please give a separate score for criteria.

1. **Significance** Should this research be done?

Does this project advance our knowledge and understanding of achieving health equity?

Your score? ____5_______. Between 1- 9 with 1 being the best score.

Strengths

- Prior research reviewed is fairly detailed and comprehensive.

Weaknesses

- Although much emphasis is on the comprehensive or complex nature of the various pieces of data collected, it is not clear whether prior research has looked at the joint contribution of the factors mentioned.
- No conceptual framework is identified.

2. **Investigator(s).** Will it be done?

Is the team qualified to accomplish the tasks identified? Do they have relevant background and experience? Remember this funding is intended for early-stage investigators who may not have a long career record but should have expertise in the area.

Your score? ____3_______. Between 1- 9 with 1 being the best score.

Strengths

- The collaborative team has expertise in clinical care, nursing and microbiome processing and some expertise in data analysis.

Weaknesses

- Maybe more of a minor budget item, but Co-investigators have no time on the proposal.
- No data analyst is identified, and the PI appears to be in charge of analyzing the data for both primary data and microbiome piece.

3. **Innovation.** Is the idea sufficiently novel, that it should be done?

Will the methods or outcomes provide new information?

Your score? ____5_______. Between 1- 9 with 1 being the best score.

Strengths

- The combination of factors being collected in the proposal is innovative.
- Only a modest amount of research on microbiome and RA outcomes has been previously done.
- There is somewhat limited research among patients with RA from diverse and underserved communities.

Weaknesses

- It is not always clear what is now known for each of the primary predictor/outcome variables, or perhaps where the gaps are or why the proposed methods are the best way to fill in this gap.

4. **Approach**. Can it be done?

Are the steps the research team is proposing understandable? Do the steps seem to make sense to address the problem identified?

Your score? ____5_______. Between 1- 9 with 1 being the best score.

Strengths

- The proposal includes a range of predictor variables shown to be important in the literature.
- The outcome measures used have been validated, both for predictor variables and outcome variables.
- The PI has shown prior experience with recruitment in similar clinic settings and has shown to be highly effective in recruitment.
- Investigators outlined proportion of residents in AZ counties by ethnicity, suggesting their sample will be more diverse than prior research (which recruited predominantly non-Hispanic White populations).
- Analyses adjust for important confounding variables such as age, gender, BMI, tobacco use and RA medication usage.
- Preliminary data is a strength and well-aligned with the current proposal.

Weaknesses

- It is not entirely clear how a cross-sectional study can answer the complex interplay between several predictor variables.
- Analyses are outlined at times as ‘linear and logistic regression models’. Although it is indicated that the outcomes are a disability questionnaire and pain scale, it is not clarified how the outcome is defined (if logistic, is there a threshold of pain or disability?). Reading the (nicely detailed) categories, an ordinal regression may be most appropriate, or if using a logistic regression, mention which categories are being compared.
- Picky, but the hypotheses state that higher …. ‘will result in’, suggesting causality, even though this is a cross-sectional study.
- The power analysis mentions an effect size of 0.15, but it is unclear whether this is in line with prior research or consistent across predictor variables (since several separate analyses are conducted.

5. **Environment.** Will it be done, thinking about the time investigators are investing and equipment available to them.

Does the research team describe resources that will ensure they can accomplish the goals of the project?

Your score?____2_______. Between 1- 9 with 1 being the best score.
**Strengths**<br>• Collaborations with community partners appear to have been well-established.<br>• There are support letters from mentors, collaborators, community partner and department chair.<br>• Resources are highlighted and strategically include TGen, PMI and UA Behavioral Measurement and Interventions Shared Resource (BMISR) for example.<br>**Weaknesses**<br>•

ADDITIONAL REVIEW CRITERIA

As applicable for the project proposed, reviewers will consider the following additional items in the determination of scientific and technical merit, but will not give separate scores for these items**.**

— Responses for Protections for Human Subjects, Vertebrate Animals, and Biohazards **are required from reviewers for all applications**.

— A response for Inclusion Plans is required from reviewers for applications proposing Human Subjects Research, except those designated Exemption 4.

Study Timeline (Specific to applications designated clinical trial on the electronic cover sheet)
**Strengths**<br>• Timeline appears reasonable<br>**Weaknesses**<br>•

Protections for Human Subjects
Acceptable Risks and/or Adequate Protections<br>Comments (Required Unless Not Applicable):<br>• Overall risks to participants are modest.<br>Data and Safety Monitoring Plan (Applicable for Clinical Trials Only):<br>Not Applicable (No Clinical Trials)<br>Comments (Required Unless Not Applicable):<br>○

Inclusion Plans **Applicable Only for Human Subjects research and not IRB Exemption #4.**
• Sex/Gender: Distribution justified scientifically<br>• Race/Ethnicity: Distribution not justified scientifically

- For NIH-Defined Phase III trials, Plans for valid design and analysis: Click Here to Select
- Inclusion/Exclusion Based on Age: Click Here to Select

Comments (Required Unless Not Applicable):

- Although the project will focus on recruitment of diverse populations, it is not indicated whether recruitment goals inlcude a goal of a proportions of each subpopulation, especially those at highest risk. However, investigators did outline proportion of residents in AZ counties by ethnicity.

Vertebrate Animals
Is the proposed research involving vertebrate animals scientifically appropriate, including the justifications for animal usage and protections for research animals described in the Vertebrate Animals section (and method of euthanasia described in the Cover Page Supplement or PHS Supplemental Form, if applicable)?<br>Not Applicable (No Vertebrate Animals)<br>Comments (Required Unless Not Applicable):<br>•

Biohazards
Not Applicable (No Biohazards)<br>Comments (Required Unless Not Applicable):<br>•

Resubmission
Comments (if applicable):<br>• N/A

Renewal
Comments (if applicable):<br>• N/A

Revision
Comments (if applicable):<br>• N/A

ADDITIONAL REVIEW CONSIDERATIONS

As applicable for the project proposed, reviewers will address each of the following items, but will not give scores for these items and should not consider them in providing an overall impact/priority score.

Applications from Foreign Organizations
Not Applicable (No Foreign Organizations)<br>Comments (Required Unless Not Applicable):<br>•

Select Agents
Not Applicable (No Select Agents)<br>Comments (Required if Unacceptable):<br>•

Resource Sharing Plans
Not Applicable (No Relevant Resources)<br>Comments (Required if Unacceptable):<br>•

Authentication of Key Biological and/or Chemical Resources
Not Applicable (No Relevant Resources)<br>Comments (Required if Unacceptable):<br>• *Don't think the microbiome fecal samples fall under this category

Budget and Period of Support
Budget Modifications Recommended (in amount/time)<br>Recommended budget modifications or possible overlap identified:<br>• Only the PI has time allocated to the proposal. it is not indicated whether in-kind contributions will be made by co-investigators. The budget for microbiome sampling appears very modest.

SHERC Pilot Project Proposal Review

Application Title: Is it a gut feeling? The impact of socioeconomic status, diet quality, and gastrointestinal health on disease activity in rheumatoid arthritis.

Principal Investigator(s): Maureen McGarrity-Yoder

Reviewer #: 3

OVERALL IMPACT

Please provide an **overall impact score** to reflect your assessment of the likelihood for the project to yield a sustained, meaningful contribution to the research field(s) involved. Prior to providing your Overall score, please provide a score and develop bulleted strengths and weaknesses statements for the five criteria: Significance, Investigators, Innovation, Approach and Environment. If you do not have comments for the strengths and weakness, stating *None noted* is acceptable.

Overall Impact *Write a paragraph summarizing your review. What is your overall impact score?______4_____. Between 1- 9 with 1 being the best score and 5 being average.*
This study proposed to address a significant knowledge gap in the impact of socioeconomic status, diet quality, gut microbiome composition and gastrointestinal inflammation on disease activity in adults with Rheumatoid arthritis (RA). This is a cross-sectional, descriptive study that will include 78 adults with RA. This study will contribute towards providing preliminary data to support the submission of an R21 NIH grant for a dietary intervention to support a healthy GI and decrease RA inflammation at the end of the proposed study. Overall, this is a clearly written proposal by a PI with some experience in the area of research being proposed. Weaknesses noted are the lack of methodological details on the timing of collection, storage, and of fecal samples collected. Additionally, data management and the bioinformatics aspect of the proposed study were not addressed.

Impact	Score	Descriptor	Additional Guidance on Strengths/Weaknesses
High	1	Exceptional	Exceptionally strong with essentially no weaknesses
	2	Outstanding	Extremely strong with negligible weaknesses
	3	Excellent	Very strong with only some minor weaknesses
Medium	4	Very Good	Strong but with numerous minor weaknesses
	5	Good	Strong but with at least one moderate weakness
	6	Satisfactory	Some strengths but also some moderate weaknesses
Low	7	Fair	Some strengths but with at least one major weakness
	8	Marginal	A few strengths and a few major weaknesses
	9	Poor	Very few strengths and numerous major weaknesses

SCORED REVIEW CRITERIA

Please consider each of the five review criteria below to determine merit. Please give a separate score for criteria.

1. **Significance** Should this research be done?

Does this project advance our knowledge and understanding of achieving health equity?

Your score? _____4______. Between 1- 9 with 1 being the best score.

Strengths

- Strong rationale for the importance of the topic and project has high relevance to SHERC goals.
- Not all individuals with RA may reach remission and several factors may have an impact, therefore examining other factors including social determinants is warranted.

Weaknesses

- Worsened patient outcomes may be seen in Hispanics/Latinos and unfortunately may not be able to participate in the study.
- How diet and cultural differences in the diet would be addressed in the collection of data and analyses were not fully addressed.

2. **Investigator(s).** Will it be done?

Is the team qualified to accomplish the tasks identified? Do they have relevant background and experience? Remember this funding is intended for early-stage investigators who may not have a long career record but should have expertise in the area.

Your score? ____3_______. Between 1- 9 with 1 being the best score.

Strengths

- PI is very well suited to the study has relevant expertise, experience working with the study sample, and a great collaborative network.

- The team of investigators have the required skills and knowledge to conduct the proposed study.
- The team have previously worked together.

Weaknesses

- Letter of support for recruitment was not provided by the University of Arizona Arthritis Center and Arizona Arthritis and Rheumatology Associates offices.
- Who will assist with the bioinformatics aspect of the study was not addressed.

3. **Innovation.** Is the idea sufficiently novel, that it should be done?

Will the methods or outcomes provide new information?

Your score? _____4______. Between 1- 9 with 1 being the best score.

Strengths

- First study to consider associations between SES, diet quality, gut microbiome composition, GI tract inflammation, and disease activity in adults with RA at the same time and in combination.
- Gut microbiome study in RA and measurement of Calprotectin.

Weaknesses

- Existing dietary tool not considered novel.

4. **Approach**. Can it be done?

Are the steps the research team is proposing understandable? Do the steps seem to make sense to address the problem identified?

Your score? _____5______. Between 1- 9 with 1 being the best score.

Strengths

- Inclusion of diet in a gut microbiome study and use of the expertise of a registered dietitian.
- Experience enrolling participants with RA from the proposed sources.
-

Weaknesses

- The timing of sample collection is a concern, especially it is not clear how long participants will have to mail their samples and some of the data will come from medical records.
- Who will collect the food frequency questionnaire and are they trained? Why was the food frequency questionnaire chosen over 24 hour recalls?
- Storage conditions of the samples collected, batch effects, contamination, and whether negative and positive samples will be used to assess contamination were not addressed.
- In some counties it is expected that Hispanics make up about 30% of the population where the RA clinics are located. The proposal states that the PI will allow for diverse recruitment, however the study will only enroll English speaking participants.

- Ethnicity can affect cultural eating habits and therefore may influence the gut microbiome and play in role in the differences observed. How this may affect the analyses were not addressed.
- Is the food frequency tool validated for the collection of data from different ethnic groups?
- The proposal includes gut diversity as a predictor variable. Different measures exist to estimate gut microbiome diversity and which measures will be used was not included.
- DNA extraction, sequencing-related, and relative abundances determination methods should be described.
- Bioinformatics and data management was not described in the proposal. Quality control methods should be included that will filter or remove reads and samples.
- How recent antibiotic use by the participant will be handled in the analysis or design was not provided.
- Sex as a biological variable was not addressed in the proposal.

5. **Environment.** Will it be done, thinking about the time investigators are investing and equipment available to them.

Does the research team describe resources that will ensure they can accomplish the goals of the project?

Your score?_____3______. Between 1- 9 with 1 being the best score.

Strengths

- The infrastructure described seems to be able to support the PI in the conduct of the study.
- The study will provide a strong foundation of team science and mentoring for the PI.

Weaknesses

- Storage capabilities were not included.
- Data management and software availability were not described.

ADDITIONAL REVIEW CRITERIA

As applicable for the project proposed, reviewers will consider the following additional items in the determination of scientific and technical merit, but will not give separate scores for these items**.**

— Responses for Protections for Human Subjects, Vertebrate Animals, and Biohazards **are required from reviewers for all applications**.

— A response for Inclusion Plans is required from reviewers for applications proposing Human Subjects Research, except those designated Exemption 4.

Study Timeline (Specific to applications designated clinical trial on the electronic cover sheet)

Strengths

- Dissemination of findings and R21 grant activities included.

Weaknesses

- Which funding agency and funding announcements will be targeted not included.
- Batch-effects for analysis of specimens may occur as analysis is spread over 4 months.

Protections for Human Subjects
Acceptable Risks and/or Adequate Protections<br>Comments (Required Unless Not Applicable):<br>•<br>Data and Safety Monitoring Plan (Applicable for Clinical Trials Only):<br>Not Applicable (No Clinical Trials)<br>Comments (Required Unless Not Applicable):<br>○

Inclusion Plans **Applicable Only for Human Subjects research and not IRB Exemption #4.**
• Sex/Gender: Distribution not justified scientifically<br>• Race/Ethnicity: Distribution justified scientifically<br>• For NIH-Defined Phase III trials, Plans for valid design and analysis: Not applicable<br>• Inclusion/Exclusion Based on Age: Distribution justified scientifically<br>Comments (Required Unless Not Applicable):<br>• Exclusion by Spanish speaking may reduce the inclusion of Hispanic/Latinos at risk

Vertebrate Animals
Is the proposed research involving vertebrate animals scientifically appropriate, including the justifications for animal usage and protections for research animals described in the Vertebrate Animals section (and method of euthanasia described in the Cover Page Supplement or PHS Supplemental Form, if applicable)?<br>Not Applicable (No Vertebrate Animals)<br>Comments (Required Unless Not Applicable):<br>•

Biohazards
Acceptable<br>Comments (Required Unless Not Applicable):

•

Resubmission
Comments (if applicable):<br>•

Renewal
Comments (if applicable):<br>•

Revision
Comments (if applicable):<br>•

ADDITIONAL REVIEW CONSIDERATIONS

As applicable for the project proposed, reviewers will address each of the following items, but will not give scores for these items and should not consider them in providing an overall impact/priority score.

Applications from Foreign Organizations
Not Applicable (No Foreign Organizations)<br>Comments (Required Unless Not Applicable):<br>•

Select Agents
Not Applicable (No Select Agents)<br>Comments (Required if Unacceptable):<br>•

Resource Sharing Plans
Not Applicable (No Relevant Resources)<br>Comments (Required if Unacceptable):<br>•

Authentication of Key Biological and/or Chemical Resources
Not Applicable (No Relevant Resources)<br>Comments (Required if Unacceptable):

•

Budget and Period of Support
Recommend as Requested<br>Recommended budget modifications or possible overlap identified:<br>•
